# Multicellular tumor spheroids in microcapsules as a novel 3D in vitro model in tumor biology

**DOI:** 10.1186/1753-6561-7-S6-P116

**Published:** 2013-12-04

**Authors:** Elena Markvicheva, Daria Zaytseva-Zotova, Roman Akasov, Sergey Burov, Isabelle Chevalot, Annie Marc

**Affiliations:** 1 Shemyakin-Ovchinnikov Inst Bioorg Chem Rus Acad Sci, 117997 Moscow, Russia; 2 Institute of Macromolecular Compounds Rus Acad Sci, 199004 St-Petersburg, Russia; 3 CNRS, Laboratoire Réactions et Génie des Procédés, UMR 7274, Université de Lorraine, Vandoeuvre-lès-Nancy Cedex, 54505, France

## Background

Advantages of microencapsulation as a 3D growth system are chemically and spatially defined 3D network of extracellular matrix components, cell-to-cell and cell-to-matrix interactions governing differentiation, proliferation and cell function in vivo. The study is aimed at i) optimization of techniques for preparing microcapsules; ii) generation of multicellular tumor spheroids (MTS) by culturing tumor cells in the microcapsules; iii) study of anticancer treatment effects for both photodynamic therapy (PDT) and anti-cancer drug screening. The model allows to estimate drug doses or parameters for PDT *in vitro *before carrying out preclinical tests, and thereby to reduce a number and costs of experiments with animals commonly used.

## Materials and methods

To form MTS, tumor cell lines (mouse melanoma cells M3, human breast adenocarcinoma cells MCF-7, mouse myeloma Sp2/0 cells, human CCRF-CEM and CEM/Cl cell lines, HeLa) were encapsulated in polyelectrolyte microcapsules (200-600 μm), and cultivated for 3-4 weeks [1]. Microcapsules were fabricated from alginate (polyanion) and various polycations, namely natural polymers (modified chitosan, DEAE-dextran etc) and novel smart co-polymers (e.g. chitosan-graft-polyvinyl alcohol copolymers) synthesized by a Solid-State Reactive Blending technique [2]. The copolymers were characterized by FTIR, GPC and elemental analysis.

## Results

MTS based MCF-7 cells were prepared and used to study effects of PDT. To study the effect of irradiation parameters on cell viability, 2 photosensitizers (PS), namely photosense and chlorine e6 were used. Phototoxicity of PS depended on PS concentration and light energy density in both monolayer culture (MLC) and MTS. Study of cell morphology in MLC and MTS before and after PDT revealed that light energy density increase within the range of 30-70 J/cm2 resulted in cell apoptosis. However, cell survival in MTS was much higher than this in the MLC. MTS were also used to test some antitumor therapeutics (methotrexate, doxorubicin and their derivatives). An enhanced cell resistance in MTS compared to MLC both for normal and Dox-resistant cells (MCF-7, MCF-7/DXR, respectively) were observed. MTS were also proposed to evaluate cytotoxicity not only of novel therapeutics but also nanosized drug delivery systems (liposomes, micelles, nanoparticles and nanoemulsions).
